# Simple and Selective HPLC-UV/Vis Bioanalytical Method to Determine Aluminum Phthalocyanine Chloride in Skin Permeation Studies

**DOI:** 10.1155/2018/7423764

**Published:** 2018-02-01

**Authors:** Thaiene Avila Reis, Ana Elise Jaculi, Rubens da Costa Alves, Tais Gratieri, Ricardo Bentes Azevedo, Graziella Anselmo Joanitti, Guilherme Martins Gelfuso, Marcilio Cunha-Filho

**Affiliations:** ^1^Laboratory of Food, Drugs and Cosmetics (LTMAC), School of Health Sciences, University of Brasília, 70.910-900 Brasília, DF, Brazil; ^2^Department of Genetics and Morphology, Institute of Biological Sciences, University of Brasília, 70.910-900 Brasília, DF, Brazil; ^3^Faculty of Ceilandia, University of Brasília, 72.220-900 Brasília, DF, Brazil

## Abstract

Considering the feasibility of the aluminum phthalocyanine chloride (AlPcCl) application in the topical photodynamic therapy of cutaneous tumors and the lack of HPLC methods capable of supporting skin permeation experiments using this compound, the aim of this study was to obtain a simple and selective chromatographic method for AlPcCl determination in skin matrices. A HPLC-UV/Vis method was developed using a normal-phase column operating at 30°C, an isocratic mobile phase of methanol : phosphoric acid (0.01 M) at 1.5 mL/min, and detection at 670 nm. The method exhibited (i) selectivity against various contaminants found in the different skin layers, (ii) high drug extraction capacity from the hair follicle (>70%) and remaining skin (>80%), and (iii) low limits of detection and of quantification (0.03 and 0.09 *μ*g/mL, resp.). The method was also linear in the range from 0.1 to 5.0 *µ*g/mL (*r* = 0.9994) and demonstrated robustness with regard to experimental chromatographic parameters according to a factorial design. Lastly, the developed method was successfully tested in in vitro skin permeation studies of AlPcCl, proving its effectiveness in the development of pharmaceutical delivery systems containing this drug for topical photodynamic therapy of skin cancers.

## 1. Introduction

Photodynamic therapy has been clinically investigated for topical treatment of several types of skin cancers such as Bowen's disease, actinic keratosis, and melanomas [1, 2]. In this kind of treatment, a photosensitizing agent is administered to the patient through the skin or mucosae followed by photoirradiation of exposed areas, producing reactive oxygen species that inhibit the growth of tumoral cells with great specificity [1]. This therapeutic approach presents several advantages over the traditional cancer treatments (surgery, radiotherapy, and chemotherapy), including more effectiveness and reduction of side effects [2].

Aluminum phthalocyanine chloride (AlPcCl) is a second-generation photosensitizing agent with potent antitumor action and high affinity to cancer tissues [3]. Despite the high photodynamic efficiency on tumor treatment, AlPcCl is highly hydrophobic, which hinders its skin absorption, leading to low bioavailability in the affected areas. Moreover, AlPcCl molecules aggregate in water, forming dimers of (PcAl)_2_ that could lead to the loss of their photodynamic activity [3–5].

The exploitation of the therapeutic potential of AlPcCl demands stable aqueous delivery systems, which could increase drug aqueous solubility and facilitate the diffusion through the skin. Some attempts in this sense have been studied recently with the preparation of AlPcCl nanostructured systems [3, 4, 6, 7].

The first step to support the development of effective topical therapeutic systems containing AlPcCl involves an adequate analytical method for quantification of AlPcCl in the different skin layers. The literature describes some methods for determination of AlPcCl using spectrophotometry or chromatography based on fluorescence or mass analysis detection, which requires less assessable HPLC detectors [6, 8–10]. Moreover, to our knowledge, there are no reports of a method with required selectivity to determine AlPcCl in the skin, which is a complex biological matrix.

In this way, the aim of this study was to obtain a chromatographic method for AlPcCl determination in skin permeation studies, enabling the development of dermatological pharmaceutical systems containing this photosensitizing agent. For this, experimental design was focused on achieving selectivity against skin contaminants using a simple and fast isocratic method with UV-Vis detection.

## 2. Experimental

### 2.1. Chemical and Reagents

AlPcCl was purchased from Sigma-Aldrich (Steinheim, Germany). 2-hydroxypropyl-*β*-cyclodextrin (lot A1306A0054, substitution degree of 4.1 to 5.1, HP-*β*-CD) was kindly gifted by Ashland Specialty Ingredients (USA). Methanol, phosphoric acid, and Tween 20 were obtained from J. T. Baker (Phillipsburg, USA), Sigma-Aldrich (Steinheim, Germany), and Dinâmica Química Contemporânea (São Paulo, Brazil), respectively. The water used in all preparations was of Milli-Q grade (Millipore, Illkirch-Graffenstaden, France). All solvents and reagents were of analytical grade.

### 2.2. Method Development

AlPcCl solution in methanol at 5 *µ*g/mL was scanned at a UV-Vis spectrophotometer (Shimadzu, UV-1800) using a quartz cell with 10 mm optical path length in order to choose the appropriate wavelength for use in HPLC analysis. Different chromatographic conditions, such as different columns (a reverse-phase C_18_ and a normal-phase column), composition of mobile phase (from 60% to 80% of methanol in acidified water), and flow rate (1.0 mL/min and 1.5 mL/min), were tested, aiming to obtain AlPcCl chromatographic peaks with acceptable analytical performance.

### 2.3. Chromatographic Conditions

The HPLC systems (LC-20AD model, Shimadzu, Kyoto, Japan) used comprised two pumps (LC-20AT model), an automatic injector (9SIL-20AD model), an oven (CTO-20AS model), a spectrophotometric detector (SPD-M20A model), and a computer equipped with the analysis software Shimadzu LC. A normal-phase *µ*Porasil™ column (300 mm × 3.9 mm, 10 *µ*m) from Waters (Massachusetts, USA) was used as the stationary phase. The mobile phase consisted of methanol : phosphoric acid 0.01 M (80 : 20, v/v), which flowed isocratically at a rate of 1.5 mL/min. Oven was set at 30°C, and detection was performed at 670 nm. Sample volumes of 20 *µ*L were injected in each analysis. Stock solutions of AlPcCl (50.0 *µ*g/mL) were prepared by dissolving 2.5 mg of the drug in 50 mL of methanol, and the work solutions were prepared from the stock solutions by proper dilution with methanol. This solvent was selected based on a preliminary solubility study that has shown that AlPcCl solubility in methanol, ethanol, and 5% Tween 20 aqueous solution was 9.9 ± 2.8 mg/mL, 5.7 ± 0.3 mg/mL, and 0.7 ± 0.4 mg/mL, respectively.

### 2.4. Method Validation

The analytical method was validated according to the current health regulations for quality control of medicines [11], considering the parameters of selectivity, linearity, limit of detection (LOD), limit of quantification (LOQ), precision, accuracy, and robustness.

#### 2.4.1. Selectivity

First, the skin contaminants stratum corneum (SC), hair follicles (HFs), and the remaining skin obtained following the differential tape stripping of fragments of porcine ear skin (1 cm^2^), as described before [12], were prepared. Briefly, each skin layer (SC, HF, and the remaining skin) was placed in individual closed glass flasks with 5 mL of methanol and left overnight under stirring (300 rpm) and then filtered through a 0.22 *μ*m membrane.

The capacity of a method to distinguish and quantify AlPcCl unequivocally in the presence of interferences was evaluated using skin contaminants, as well as the solubilizing agent HP-*β*-CD. Samples containing AlPcCl in 5.0 *µ*g/mL were prepared, with and without each of the contaminants. The assays were performed in sextuplicate, and the results of the peak area were analyzed following the one-way analysis of variance (ANOVA) with a significance level of 0.05.

#### 2.4.2. Robustness

The robustness of the method was assessed with the performance of intentional variations in three important chromatographic parameters of analysis following a factorial design 2^3^ [13]. The selected parameters were oven temperature (Temp), flow rate (FR), and acid concentration of the mobile phase (Acid). They were tested in the range of 3–10% as described in Table 1. All assays were performed with AlPcCl samples at 2.5 *µ*g/mL in triplicate in a randomized order. A prediction equation for each peak area was proposed using the analysis of multiple regressions stepwise. The model was validated following the ANOVA with a significance level of 0.05. All statistical calculations were performed using the software Design-Expert, version 9 (Minneapolis, USA).

#### 2.4.3. Linearity

A calibration curve was built with AlPcCl solutions in the range of 0.1 *μ*g/mL to 5 *μ*g/mL using six levels of concentration randomly evaluated in triplicate. Data were fitted using linear least-squares regression. Student's *t*-test (*p*=0.05) was employed to calculate the angular coefficient significance followed by proportionality tests, whereas the response factors were calculated by the ratio between the peak area and AlPcCl concentration [14]. The residues were estimated based on the difference between theoretical and experimental values, which were calculated from the calibration curve.

#### 2.4.4. Limit of Detection (LOD) and Limit of Quantification (LOQ)

The theoretical LOD and LOQ were calculated from the calibration curve according to the following equations:

(1) $$\begin{array}{c} \text{LOD}=3.3\times \frac{\sigma }{S}, \end{array}$$

(2) $$\begin{array}{c} \text{LOQ}=10\times \frac{\sigma }{S}, \end{array}$$

where *σ* is the standard deviation of *y*-axis interception values and *S* is the angular coefficient.

#### 2.4.5. Precision

Two levels of precision were evaluated: repeatability and intermediate precision. Repeatability (intra-assay) was verified for three concentrations of AlPcCl (1.0, 2.5, and 5.0 *μ*g/mL) using three replicates. Intermediate precision (interassay) was evaluated on two different days, using two different HPLC systems and two different analysts at AlPcCl concentrations of 1.0, 2.5, and 5.0 *μ*g/mL in triplicate each. Results of precision were expressed as coefficient of variation in each assay condition (CV) and in all levels of variation for each drug concentration (overall CV).

#### 2.4.6. Accuracy

The accuracy was evaluated based on the percentage of AlPcCl recovery from the skin matrices (SC, HF, and the remaining skin). First, the layers of fragments of the skin were separated following the differential tape stripping technique. Each skin layer was dropped with different volumes of a methanolic solution of AlPcCl, which corresponded to 5.0, 12.5 *μ*g, and 25.0 *μ*g of the drug. After evaporation of the solvent, skin samples were soaked in 5 mL of methanol, and extraction was conducted for 1 h under stirring (1,000 rpm) at room temperature. Afterwards, samples were filtered and analyzed using the proposed method. AlPcCl recovery was determined from the ratio of the drug amount extracted from the skin samples to the AlPcCl amount added to each skin layer. The analyses were performed with three samples of each evaluated final concentration (1.0, 2.5, and 5.0 *μ*g/mL). Results of accuracy were calculated in terms of recovery (*R*) according to (3):

(3) $$\begin{array}{c} R=\left(\frac{\text{measured concentration}}{\text{nominal concentration}}\right)\times 100. \end{array}$$

### 2.5. Application of the Method in Skin Permeation Studies

The analytical method developed and validated was used to quantify AlPcCl from skin layers after permeation studies. Thus, permeation experiments were carried out in vitro using modified Franz-type diffusion cells mounted with excised porcine skin separating the donor and receptor compartments. The receptor compartment was filled with aqueous solution of 5% (w/v) Tween 20, which was magnetically stirred at 500 rpm and maintained at 32°C. 1 mL of the formulation (15.0 *μ*g/mL of AlPcCl in aqueous solution with 20% HP-*β*-CD, pH 5) was placed in donor compartments and left to permeation for 6 h and 12 h. After this period, the skin was removed from diffusion cells and cleaned with fresh water, and AlPcCl was extracted from each skin layer and properly quantified. The permeation experiment was performed in quintuplicate.

## 3. Results and Discussion

### 3.1. Optimization of Chromatographic Conditions

The UV-Vis spectrum of AlPcCl showed two peaks of maximum absorption at 354 nm and 670 nm. The wavelength of 670 nm showed less absorption for analytical interferences, especially for the skin contaminants, and was, therefore, selected for the subsequent studies. The main tests carried out for the phase of method development are summarized in Table 2.

The chromatographic conditions used as a starting point were based on the methods described in the literature, which used fluorescence detection [3]. In these conditions (trials 1 and 2), an asymmetric peak of the analyte was obtained, with elution very close to the solvent, in about 2.7 min.

A normal-phase column with a longer length was tested in an attempt to increase AlPcCl retention time and to improve peak performance. Subsequent modifications in the mobile-phase composition with reduction of its polarity increased drug retention time (trials 3–6). After this, a last change in the flow rate was able to adjust the AlPcCl retention time to 3.71 min (trial 7). In this way, the final chromatographic conditions produced a symmetrical peak of the analyte with suitable chromatographic parameters, including adequate retention time.

### 3.2. Validation

An imperative requirement to enable the method use in skin permeation studies is to prove its selectivity against the skin tissue interferences. In addition, its selectivity against the solubilizing agent HP-*β*-CD was also performed since the drug encapsulation could modify physicochemical parameters of the guest molecule [15].

**Figure 1:**
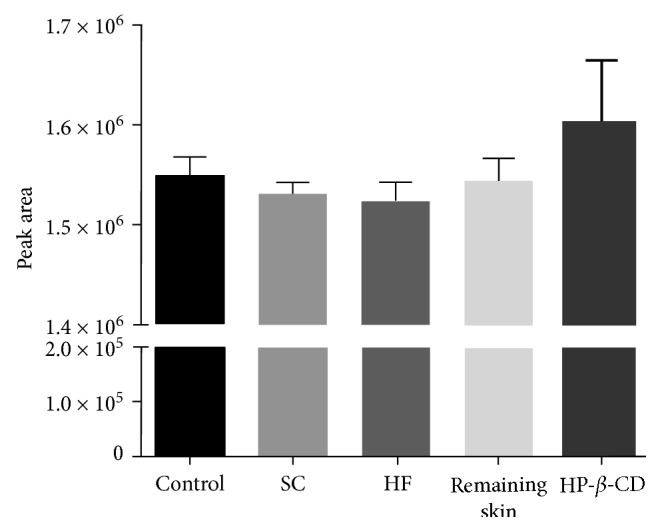
Selectivity of the method for AlPcCl determination at 5.0 *μ*g/mL alone and with the interferences SC, HF, and the remaining skin and HP-*β*-CD.

No significant difference occurred between peak areas of AlPcCl eluted alone or in combination with interferences (Figure 1). Moreover, no changes in retention time or in chromatographic performance parameters were observed, demonstrating the absence of interaction between the components during the analyses. Thus, the method proved to be selective to assess the analyte in the presence of skin components or HP-*β*-CD.

It was then evaluated how variations in chromatographic parameters can interfere in the analyte peak area to assess the robustness of the method. Oscillations in acid concentration of the mobile phase or oven temperature did not cause any statistical effect in this response (*p*=0.8163 and *p*=0.9307, resp.). Flow rate, on the other hand, was decisive for the analytical response (*p* < 0.0001). Indeed, this factor alone is responsible for more than 90% of the response, while the random error explains the remaining 9%.

**Figure 2:**
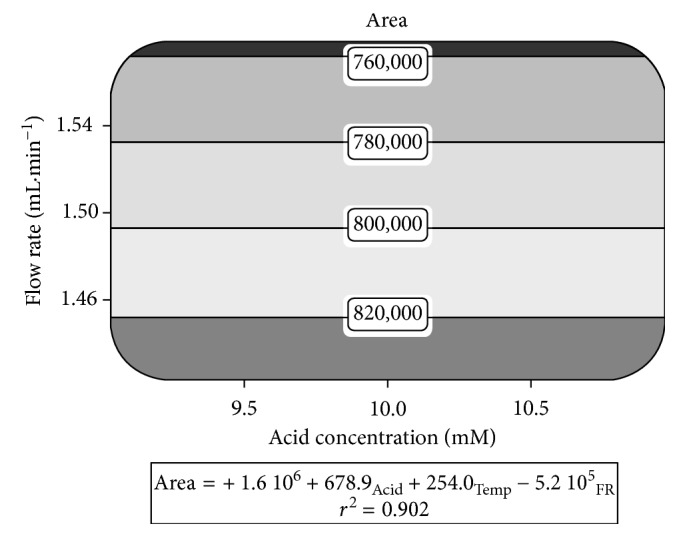
Response surfaces for the AlPcCl peak area in robustness assay together with predictive equations and *r* ^2^. Dark areas show regions with more intense changes in these responses.

In response surface graph shown in Figure 2, peak area variation up to 8% is observed according to the flow rate. Similar effect is commonly described in the literature for other chromatographic methods [14, 16, 17], evidencing the importance of maintaining this parameter controlled and to perform periodical maintenance in the pumps. The predictive equation fitted a linear model and showed a high extrapolative capacity (*r* ^2^ = 0.902). The negative coefficient for the flow rate factor showed that a larger flow rate leads to a reduction in the sensitivity of the method (Figure 2).

Linear regression provided the equation *y* = 306,119*x* + 4,219.1, where *y* is the peak area and *x* is the AlPcCl concentration in *µ*g/mL. The correlation coefficient close to one (0.9994) met the validation requirements (minimum of 0.999 [11]), proving the method capacity to provide proportionality between the area and concentration values in a large range of concentrations (0.1–5.0 *µ*g/mL).

The angular coefficient was different of zero according to Student's *t*-test and its high numerical value of 306, 119 indicated appropriate response of the method against changes in concentration. The proportionality test calculated following the *t*-test proved that the curve passes through the origin of coordinates, which indicates the adequacy of the concentration range used (confidence interval of −13,470 to 21,908) [16]. Residues showed approximately the same absolute value and a random data distribution without tendency.

The LOD and LOQ were calculated as 0.03 and 0.09 *μ*g/mL, respectively. The reduced values of these limits indicated the high sensitivity of the method, which is particularly interesting in this case, considering the potent effect of AlPcCl used in very low doses in photodynamic therapy [7]. Accordingly, LOD and LOQ should be appropriated to determine AlPcCl in future pharmaceutical formulation assays, including the initial points of the skin permeation kinetic studies.

The results of intra-assay precision exhibited CV below 5.0% in all of the three concentrations tested (Table 3). Interassay precision presented consistent responses for variation of the analyst, equipment, and day of analysis, with CV up to 7.8% for the lower concentration and less than 2% for the other concentrations (Table 3). This result is in accordance with recommended limits of 15% established for bioanalytical methods [17].

Considering that the lipophilic AlPcCl could interact differently with the substances that compose the different layers of the skin, which is a complex tissue, the process of extracting the drug from SC, as well as the HF and the remaining skin, was verified by the method accuracy. In this way, AlPcCl recovered from skin layers is presented in Table 4.

The developed method showed a high recovery capacity of AlPcCl in the remaining skin, with values higher than 80% for the three levels of concentrations evaluated (Table 4). Similar performance was achieved in HF, which showed values greater than 70%. Both results can be considered adequate recovery performances for determinations in biological matrices [14, 18–20].

In contrast, drug recovery from SC was low (around 50% in all concentration levels tested), caused by the high affinity of the lipophilic drug with also the lipophilic SC. In this case, the low values of CV (below 5%) endorse the use of a correction factor for the quantification of AlPcCl from this specific skin layer. This procedure is in accordance with the FDA guideline in cases of consistent, accurate, and reproducible recovery results [21].

### 3.3. Skin Permeation Experiments

**Figure 3:**
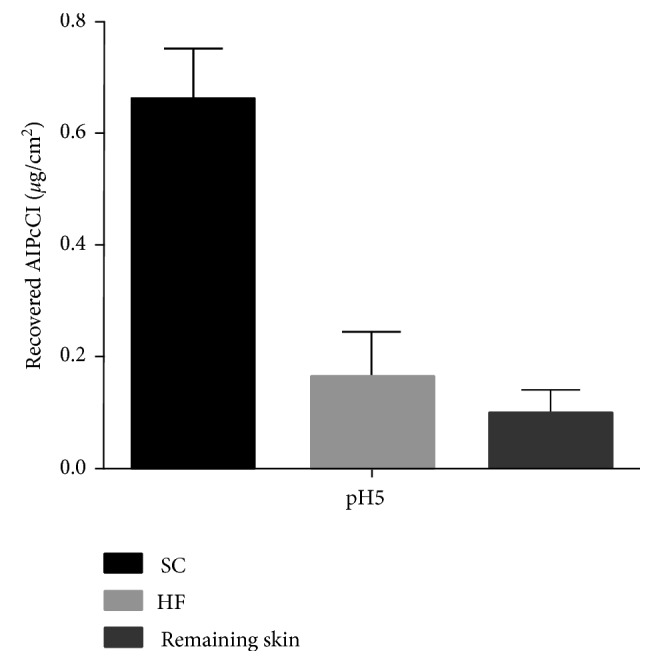
Recovered AlPcCl from porcine skin tissues after 12 h of in vitro permeation experiments using modified Franz-type diffusion cells.

An in vitro permeation study was carried out using an aqueous solution of AlPcCl as a formulation in order to verify the practical application of the developed analytical methodology for assays involving skin absorption of the drug (Figure 3). As expected, due to the high AlPcCl hydrophobicity, almost the entire dose of the drug was retained in the first skin layer, that is, the SC. Small amounts of AlPcCl were detected in the HF and remaining skin; however, those concentrations were below the LOQ of the validated method.

These results exhibit an evident technological challenge to be overcome through the development of drug delivery systems that could improve AlPcCl skin penetration. Accordingly, the validated method proved to be capable of giving support for such a formulation development.

## 4. Conclusion

A simple and rapid HPLC-UV/Vis method for determination of AlPcCl was developed using a normal stationary phase and an isocratic, organic mobile phase. The method showed (i) selectivity against diverse contaminants found in the different skin layers, (ii) high drug extraction capacity from the HF and remaining skin, (iii) low limits of detection and quantification, and (iv) compliance with the other validation parameters established for methods used in quality control of medicines. Lastly, the developed method was successfully tested in in vitro skin permeation studies of AlPcCl, proving its benefit in the development of topical delivery systems containing this drug to enable topical photodynamic therapy of skin cancers.

## Figures and Tables

**Table 1:** Experimental design for robustness assays.

Experiment	Temp (°C)	FR (mL/min)	Acid (mM)
1	29	1.425	0.009
2	31	1.425	0.009
3	29	1.575	0.009
4	31	1.575	0.009
5	29	1.425	0.011
6	31	1.425	0.011
7	29	1.575	0.011
8	31	1.575	0.011

Temp: oven temperature; FR: flow rate; Acid: phosphoric acid concentration of the mobile phase.

**Table 2:** Optimization of chromatographic conditions for AlPcCl determination.

Trial	Column	Mobile phase (v : v)	Flow rate (mL/min)	Tailing factor	Resolution	Retention time (min)	Peak characteristics
1	RP C_18_, 15 cm	MeOH : H_3_PO_4_ 0.01 M (60 : 40)	1.0	—	3.104	2.7	Fronting peak
2	RP C_18_, 15 cm	MeOH : H_3_PO_4_ 0.01 M (63 : 37)	1.0	0.813	2.954	2.7	Fronting peak
3	NP, 30 cm	MeOH : H_3_PO_4_ 0.01 M (60 : 40)	1.0	1.065	1.860	2.5	Early peak
4	NP, 30 cm	MeOH : H_3_PO_4_ 0.01 M (70 : 30)	1.0	0.947	3.143	2.89	Early peak
5	NP, 30 cm	MeOH : H_3_PO_4_ 0.01 M (75 : 25)	1.0	0.901	3.881	3.21	Early peak
6	NP, 30 cm	MeOH : H_3_PO_4_ 0.01 M (80 : 20)	1.0	0.908	5.070	4.77	Suitable peak
7	NP, 30 cm	MeOH : H_3_PO_4_ 0.01 M (80 : 20)	1.5	0.876	4.475	3.71	Suitable peak

**Table 3:** Intra- and interassay precision of the AlPcCl HPLC method.

Theoretical concentration (*μ*g/mL)			Experimental concentration (*μ*g/mL)	CV (%)	Overall CV (%)
1.0	Analyst 1	Day 1	1.00	1.49	7.80
	Equipment 1	Day 2	1.03	2.71	
	Analyst 2	Day 1	0.88	3.74	
	Equipment 2	Day 2	0.83	3.07	
2.5	Analyst 1	Day 1	2.49	1.12	1.72
	Equipment 1	Day 2	2.54	4.33	
	Analyst 2	Day 1	2.49	0.88	
	Equipment 2	Day 2	2.49	1.94	
5.0	Analyst 1	Day 1	5.05	1.39	1.66
	Equipment 1	Day 2	5.03	4.26	
	Analyst 2	Day 1	4.53	2.58	
	Equipment 2	Day 2	4.48	1.82	

CV: coefficient of variation.

**Table 4:** Recovery of the analytical method for determination of AlPcCl from skin layers.

Theoretical concentration of AlPcCl (*µ*g/mL)	SC		HF		Remaining skin	
	AlPcCl recovered (%)	CV (%)	AlPcCl recovered (%)	CV (%)	AlPcCl recovered (%)	CV (%)
1.0	50.3	4.97	76.4	8.94	84.3	1.99
2.5	44.3	4.78	71.1	2.97	84.2	3.90
5.0	49.9	2.21	74.3	4.26	88.5	3.70

SC: stratum corneum; HF: hair follicle; CV: coefficient of variation.

## References

[1] Lee P. K., Kloser A. (2013). Current methods for photodynamic therapy in the US: comparison of MAL/PDT and ALA/PDT. *Journal of Drugs in Dermatology*.

[2] Westers-Attema A., Lohman B. G. P. M., van Den Heijkant F. (2015). Photodynamic therapy in Bowen’s disease: influence of histological features and clinical characteristics on its success. *Dermatology*.

[3] Muehlmann L. A., Ma B. C., Longo J. P. F., Almeida Santos M. F., Azevedo R. B. (2014). Aluminum-phthalocyanine chloride associated to poly(methyl vinyl ether-comaleic anhydride) nanoparticles as a new third-generation photosensitizer for anticancer photodynamic therapy. *International Journal of Nanomedicine*.

[4] de Paula C. S., Tedesco A. C., Primo F. L., Vilela J. M. C., Andrade M. S., Mosqueira V. C. F. (2013). Chloroaluminium phthalocyanine polymeric nanoparticles as photosensitisers: photophysical and physicochemical characterisation, release and phototoxicity in vitro. *European Journal of Pharmaceutical Sciences*.

[5] Polek M., Latteyer F., Basova T. V. (2016). Chemical reaction of polar phthalocyanines on silver: chloroaluminum phthalocyanine and fluoroaluminum phthalocyanine. *Journal of Physical Chemistry C*.

[6] Muehlmann L. A., Rodrigues M. C., Longo J. P. F. (2015). Aluminium-phthalocyanine chloride nanoemulsions for anticancer photodynamic therapy: development and in vitro activity against monolayers and spheroids of human mammary adenocarcinoma MCF-7 cells. *Journal of Nanobiotechnology*.

[7] Rocha M. S., Lucci C. M., Longo J. P. (2012). Aluminum-chloride-phthalocyanine encapsulated in liposomes: activity against naturally occurring dog breast cancer cells. *Journal of Biomedical Nanotechnology*.

[8] Kyriazi M., Alexandratou E., Yova D., Rallis M., Trebst T. (2008). Topical photodynamic therapy of murine non-melanoma skin carcinomas with aluminum phthalocyanine chloride and a diode laser: pharmacokinetics, tumor response and cosmetic outcomes. *Photodermatology, Photoimmunology & Photomedicine*.

[9] Oliveira L. T., Garcia G. M., Kano E. K., Tedesco A. C., Mosqueira V. C. (2011). HPLC-FLD methods to quantify chloroaluminum phthalocyanine in nanoparticles, plasma and tissue: application in pharmacokinetic and biodistribution studies. *Journal of Pharmaceutical and Biomedical Analysis*.

[10] Siqueira-Moura M. P., Primo F. L., Peti A. P., Tedesco A. C. (2010). Validated spectrophotometric and spectrofluorimetric methods for determination of chloroaluminum phthalocyanine in nanocarriers. *Pharmazie*.

[11] ICH Q2(R1) validation of analytical procedures: text and methodology.

[12] Pereira M. N., Schulte H. L., Duarte N. (2017). Solid effervescent formulations as new approach for topical minoxidil delivery. *European Journal of Pharmaceutical Sciences*.

[13] Pinho L. A. G., Sá-Barreto L. C. L., Infante C. M. C., Cunha-Filho M. S. S. (2016). Simultaneous determination of benznidazole and itraconazole using spectrophotometry applied to the analysis of mixture: a tool for quality control in the development of formulations. *Spectrochimica Acta, Part A Molecular and Biomolecular Spectroscopy*.

[14] Angelo T., Pires F. Q., Gelfuso G. M., Silva J. K. R., Gratieri T., Cunha-Filho M. S. S. (2016). Development and validation of a selective HPLC-UV method for thymol determination in skin permeation experiments. *Journal of Chromatography B*.

[15] Cunha-Filho M. S. S., Sá-Barreto L. C. L. (2007). Use of cyclodextrins to form inclusion complexes of pharmaceutical interest. *Journal of Basic and Applied Pharmaceutical Sciences*.

[16] Nunes-Ferreira R., Angelo T., da Silva S. M. M. (2017). Versatile chromatographic method for catechin determination in development of topical formulations containing natural extracts. *Biomedical Chromatography*.

[17] Angelo T., Cunha-Filho M. S. S., Gelfuso G. M., Gratieri T. (2017). Chromatographic method for clobetasol propionate determination in hair follicles and in different skin layers. *Biomedical Chromatography*.

[18] Campos P. M., Praça F. S. G., Bentley M. V. L. B. (2016). Quantification of lipoic acid from skin samples by HPLC using ultraviolet, electrochemical and evaporative light scattering detectors. *Journal of Chromatography B*.

[19] Huo Y., Liu Y. Q., Bai Z. X., Cai Q. (2014). Determination of (4E,6E,12E)-tetradecatriene-8,10-diyne-1,3-diyl diacetate in rat plasma and tissues by HPLC-UV method and their application to a pharmacokinetic and tissue distribution study. *Journal of Analytical Methods in Chemistry*.

[20] Konda R. K., Challa B. R., Chandu B. R., Chandrasekhar K. B. (2012). Bioanalytical method development and validation of memantine in human plasma by high performance liquid chromatography with tandem mass spectrometry: application to bioequivalence study. *Journal of Analytical Methods in Chemistry*.

[21] US Food and Drug Administration (2001). *Guidance for Industry: Bioanalytical Method Validation*.

